# Effectiveness of rotavirus vaccines, licensed but not funded, against rotavirus hospitalizations in the Valencia Region, Spain

**DOI:** 10.1186/s12879-015-0811-5

**Published:** 2015-02-25

**Authors:** Silvia Pérez-Vilar, Javier Díez-Domingo, Mónica López-Lacort, Sergio Martínez-Úbeda, Miguel A Martinez-Beneito

**Affiliations:** Vaccine Research, Fundación para el Fomento de la Investigación Sanitaria y Biomédica de la Comunitat Valenciana, FISABIO-Public Health, Valencia, Spain; Health Inequalities, Fundación para el Fomento de la Investigación Sanitaria y Biomédica de la Comunitat Valenciana, FISABIO-Public Health, Valencia, Spain; Ciber de Epidemiología y Salud Pública-CIBERESP, Instituto de Salud Carlos III, Madrid, Spain

**Keywords:** Rotavirus, Rotavirus vaccines, Gastroenteritis, Diarrhea, Hospitalization, Effectiveness

## Abstract

**Background:**

Although rotavirus vaccines have been licensed in Spain for over 8 years, they are not funded by its public health systems. The analysis of their effectiveness in the Valencia Region could better inform decisions about potential inclusion in the official immunization schedule. Our aim was to assess the effectiveness of Rotarix® (RV1) and RotaTeq® (RV5) against rotavirus hospitalizations.

**Methods:**

We conducted a retrospective cohort study using the region’s health care databases, among resident children aged <3 years covered by the National Health System, during January 2007-June 2012. We compared two cohorts of vaccinated children: the first included children who received at least one dose of a rotavirus vaccine, and the second included children who were not vaccinated with rotavirus vaccines but received at least one dose of a pneumococcal vaccine, another licensed but non-funded vaccine. The main outcome was rotavirus hospitalization, either laboratory-confirmed (confirmed) or codified as rotavirus (probable). Rotavirus vaccine effectiveness (RVE) by vaccine brand was assessed using Cox proportional hazards models.

**Results:**

The study included 78,281 rotavirus and 96,643 pneumococcal vaccinees. Adjusted RVE against probable or confirmed rotavirus hospitalizations was 86% (95% CI: 78-91%) and 88% (95% CI: 81-92%) for a complete series of RV1 and RV5 respectively.

**Conclusions:**

Both rotavirus vaccines were over 85% effective against rotavirus hospitalization among young children. The high effectiveness shown argues in favor of their inclusion in the official schedule. Additional information on rotavirus vaccine safety, duration of protection, and benefit-risk will also be needed to inform such deliberations.

## Background

Rotavirus is the leading cause of severe pediatric gastroenteritis: 39% (29%-45%) of hospitalized diarrhea cases worldwide during 2000–2004 were attributable to rotavirus [1]. In the Valencia Region, rotavirus is responsible for 53% of all gastroenteritis hospitalizations among children aged <5 years [2]. Younger children have a significantly higher risk of a primary infection leading to severe diarrhea, defined as diarrhea requiring hospitalization. Most serious episodes occur among children aged 3–35 months. Children who suffer natural rotavirus infection develop some immunity to disease, decreasing the severity of subsequent infections and increasing the protective effect following each infection. Natural infection may provide protection against multiple serotypes since the antibody response to natural infection appears to be heterotypic [3,4].

Two oral live-attenuated rotavirus vaccines are currently licensed: a monovalent human vaccine (RV1), (Rotarix®; GlaxoSmithKline Biologicals, Rixensart, Belgium), indicated as a two-dose series in infants between the ages of 6–12 and 24 weeks [5], and a pentavalent bovine-human reassortant vaccine (RV5), (RotaTeq®; Merck & Co., Inc., West Point, PA, USA), indicated as a three-dose series starting at 6–12 weeks and ending ≤32 weeks of age [6]. Both vaccines showed high efficacy against serotypes included in the vaccine [7,8], and high direct effectiveness in preventing rotavirus cases and hospitalizations [9-13]. Herd immunity has been suggested by studies from countries that introduced universal rotavirus vaccination [13-15].

RV1 and RV5 have been available in Spain since August 2006 and January 2007, respectively. Although institutions such as the World Health Organization, the Centers for Disease Control and Prevention, and the Pediatric Spanish Society, recommend the inclusion of rotavirus vaccination in national immunization programmes [4,16-18], rotavirus vaccines are not funded by the Spanish National Health System (NHS). Due to the incidental finding of circovirus DNA contamination in both vaccines, the Spanish Medicines Agency suspended RV5 distribution during June-November 2010, and RV1 distribution since March 2010 [19]. As of this publication, RV1 distribution remains suspended in Spain.

Our objective was to assess rotavirus vaccine effectiveness (VE), in complete and incomplete schedules, to prevent severe rotavirus gastroenteritis among children aged <3 years in the Valencia Region using electronic health care databases. Since rotavirus vaccines in Spain, a country with universal health coverage, are licensed but not funded, results of this study could better inform decisions regarding the potential inclusion of these vaccines in the official immunization schedule.

## Methods

Retrospective cohort study of Valencia Region’s children covered by the Spanish Health Care System performed using the region’s health care databases during 1^st^ January 2007- 30^th^ June 2012.

### Study setting and data sources

The Valencia Region has a population of approximately 5,000,000 inhabitants and an annual birth cohort of around 48,000 infants. Almost all the population (98.3%) is covered by the health system [20], which includes 24 pediatric hospitals. All health care users have a unique identification number that allows linking all health care databases and all medical records. The regional population-based administrative database, SIP, collects and updates demographic data, health services assignment, and use of the NHS, for both residents and non-residents.

Hospitalized cases were obtained from CMBD, the Spanish hospital discharge database [21]. In CMBD, diagnoses and procedures are collected as an assessment of medical activity and coded by trained personnel. The coding system used is the International Classification of Diseases 9th Revision, Clinical Modification (ICD-9-CM). Using CMBD is compulsory for all public hospitals. The regional microbiological surveillance network database, RedMIVA, provided rotavirus detection results; this database is linked to all Valencia’s public microbiological laboratories daily [22]. Vaccination status was obtained from SIV, the regional vaccine information system. Funded and non-funded vaccines administered in all public and some private health centers are recorded on SIV [23]. Registration procedures are the same regardless of whether vaccines are included in the official immunization schedule.

### Study population

The study included all children aged <36 months born since 1^st^ January 2007 until the end of the study period, affiliated to the NHS at six weeks of age or earlier, residents in the Valencia Region for at least four weeks, and registered in SIV as vaccinated with at least one dose of rotavirus and/or pneumococcal conjugate vaccine (PCV). Children who received at least one dose of rotavirus vaccine (having, or not, received PCV) were included in the rotavirus-vaccinated cohort, and children who received at least one dose of PCV but no rotavirus vaccine were included in the comparison cohort. As rotavirus vaccines, PCVs are licensed but not included in the official immunization schedule; they are also administered to infants from six weeks of age onwards.

Children were excluded from the study if they were vaccinated before six weeks of age, after one year of age, with less than three weeks elapsed between administrations of subsequent vaccine doses, with more than one vaccine brand, with unknown vaccine brand, with more than two doses of RV1, or with more than three doses of RV5.

The observation period began on birthdate and ended on: (1) the date of exit in SIP (regardless of the reason), (2) the date of the first rotavirus positive result, (3) the first rotavirus hospitalization (ICD-9-CM code 008.61 in any diagnosis position), (4) the end of the study period, or (5) the date prior to reaching 36 months of age, whichever occurred earlier.

### Case definition

Our outcomes were: (a) *confirmed hospitalized rotavirus cases*, defined as first hospitalization with a discharge diagnosis of intestinal infectious disease (ICD-9-CM codes 001–009) in any diagnosis position, plus a laboratory confirmed rotavirus result associated with the admission; and (b) *probable hospitalized rotavirus cases*, defined as first hospitalization with a discharge diagnosis of enteritis due to rotavirus (ICD-9-CM code 008.61) in any diagnosis position.

A laboratory confirmed rotavirus test was considered associated to the admission if the result was available within five days prior to admission and 15 days after discharge. Stool samples were assayed using techniques made available by each hospital. The decision to perform a stool test was not systematic and depended on each health department and pediatrician.

### Exposure

Vaccination status was assessed as a time-varying exposure. Eligible children were considered as vaccinated with a dose of rotavirus vaccine when at least 14 days [7] had elapsed since each dose administration. The following categories were used:

1. *Fully vaccinated* (three doses of RV5 or two doses of RV1);
2. *Partially vaccinated two doses* (two doses of RV5);
3. *Partially vaccinated one dose* (one dose of RV5 or RV1);
4. *Unvaccinated* (absence of record for rotavirus vaccination in SIV);

If one or more of the vaccine doses registered as administered did not indicate vaccine brand, the brand assumed for all doses was the one specifically mentioned for the remaining doses.

### Statistical analysis

Proportions in distribution by gender, residence status (as registered in SIP), year of birth, having health coverage within the public system at the time of data extraction, having received any vaccination by a private provider, and performance of a rotavirus laboratory test were compared using chi-square and Fisher’s exact tests.

Age at dose administration and intervals between doses were described for rotavirus vaccinees using proportions and medians.

Rotavirus VE was calculated as (1-hazard ratio) x 100%. Cox proportional hazard models with time-varying covariates were used for estimating the hazard of rotavirus hospitalization for the study groups. The model was adjusted for gender, having received any vaccination by a private provider, and zip code (random effects). Rotavirus seasonality (December-April) [24], and year of birth did not satisfy the proportional hazard assumption; therefore, the model was stratified for them solving the non-proportional issues arising [25]. Age was implicitly adjusted for in the model. Analyses were performed: (a) jointly for confirmed and probable hospitalized rotavirus cases, and (b) for confirmed hospitalized rotavirus cases only, evaluating also the effect of being partially or fully vaccinated. An additional analysis on children fulfilling the product information criteria for timing of vaccination was also carried out (maximum age at administration of the 3^rd^ dose for RV5 was 32 weeks).

In another analysis, we estimated VE by annual rotavirus epidemic season (December-April) among fully vaccinated children. The models were adjusted for gender, receipt of at least one vaccine in a private center, and zip code (random effects).

All analyses were performed by vaccine brand. Analyses were carried out using R Statistical Software (Foundation for Statistical Computing, Vienna, Austria) version 3.0.3. All tests were two-sided with a significance level of 0.05.

### Ethical considerations

The Ethics Research Committee of the Dirección General de Salud Pública/Centro Superior de Investigación en Salud Pública approved the study.

## Results

### Characteristics of the study population

**Figure 1:**
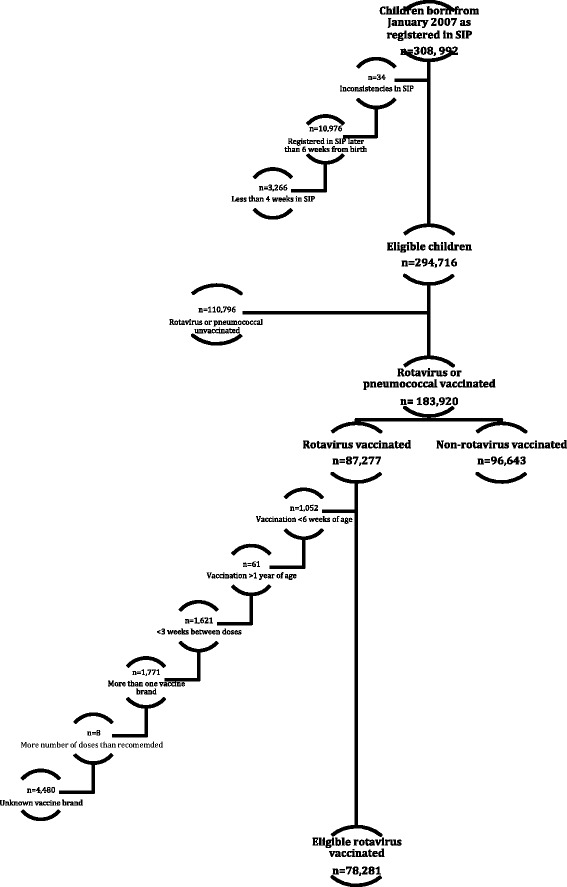
**Study population**.

The study included 174,924 eligible children, representing 59% of the 294,716 total eligible children of compatible age covered by the NHS (Figure 1). Among them, 78,281 children were vaccinated against rotavirus, contributing 173,973 person-years to the study, and 96,643 children were in the comparison group, contributing 216,416 person-years. The median follow-up was 1,013 days, with an interquartile range (IQR) of 540–1,094 days.

Almost all (96.7%) rotavirus vaccinees had also received PCVs. The cohort of children vaccinated with any rotavirus vaccine, and the comparison cohort (children vaccinated with pneumococcal vaccine) were similar with respect to gender, residence status, and performance of a rotavirus detection test during gastroenteritis hospitalization. Children included in the rotavirus cohort were more likely to be vaccinated in private centers (14.4% vs. 10.9%; p < 0.001) and slightly more likely to be covered by the universal health system (95.3% vs. 93.7%; p < 0.001) than children included in the non-rotavirus cohort. Differences in birth year between groups were found, reflecting differences in the uptake of each vaccine after their respective introduction in the Spanish market and in the start and end of suspension of each vaccine’s distribution by Spanish authorities. An apparent lower uptake of the vaccines in 2012 was due to the end of the study period in mid-2012 (Table 1).

**Table 1:** **Characteristics of study population**

	**RV1 cohort**	**RV5 cohort**	**Any rotavirus vaccine** (**RV1 or RV5**)	**Comparison cohort**
	n = 24,723	n = 53,558	n = 78,281	n = 96,643
**Gender**				
Male	12,882 (52.1%)	27,599 (51.5%)	40,481 (51.7%)	50,155 (51.9%)
Female	11,841 (47.9%)	25,959 (48.5%)	37,800 (48.3%)	46,488 (48.1%)
**Birth year** ^**a**^				
2007	3,805 (15.4%)	3,924 (7.3%)	7,729 (9.9%)	22,013 (22.8%)
2008	9,429 (38.1%)	10,092 (18.8%)	19,521 (24.9%)	16,449 (17.0%)
2009	10,759 (43.5%)	9,688 (18.1%)	20,447 (26.1%)	13,147 (13.6%)
2010	666 (2.7%)	7,476 (14.0%)	8,142 (10.4%)	25,551 (26.4%)
2011	62 (0.3%)	16,597 (31.0%)	16,659 (21.3%)	16,063 (16.6%)
2012	2 (0.01%)	5,781 (10.8%)	5,783 (7.4%)	3,420 (3.5%)
**Residence status** (**as registered in SIP**)				
Resident	24,451 (99.9%)	53,255 (99.98%)	77,706 (99.97%)	95,643 (99.9%)
Long-stay	17 (0.1%)	11 (0.02%)	28 (0.03%)	49 (0.1%)
**Covered by the universal health system** ^**b**^				
Yes	23,319 (95.3%)	50,748 (95.3%)	74,067 (95.3%)	89,633 (93.7%)
No	1,149 (4.7%)	2,518 (4.7%)	3,667 (4.7%)	6,059 (6.3%)
**Any vaccination by a private provider**				
Yes	2,846 (11.5%)	8,426 (15.7%)	11,272 (14.4%)	10,501 (10.9%)
No	21,877 (88.5%)	45,132 (84.3%)	67,009 (85.6%)	86,142 (89.1%)
**AGE** ^**c**^ **hospitalization** ^**d**^				
At least one admission	230 (0.9%)	332 (0.6%)	562 (0.7%)	1,522 (1.6%)
**Rotavirus test performed**				
During any AGE hospitalization^**d**^	183 (79.6 %)	267 (80.4 %)	450 (80.1%)	1,185 (77.9 %)

^a^Number of children born each year. The study period ended in June 2012.

^b^Answer NO refers to requested extension of the assistance, without resources/solidarity card, out-of-date accreditation, or non-accredited at the time of data extraction.

^c^Acute gastroenteritis.

^d^Discharge diagnosis of intestinal infectious disease (ICD-9-CM codes 001–009).

Of 78,281 children included in the rotavirus-vaccinated cohort, 24,723 (32%) were vaccinated with at least one dose of RV1, and 53,558 (68%) received at least one dose of RV5. A total of 21,119 (85%) and 34,865 (65%), respectively, of RV1 and RV5 vaccinees, completed the two and three-dose series. Of them, 19,751 (94%) and 30,066 (86%) of RV1 and RV5 vaccinees were vaccinated within the times indicated in the respective product information.

Among children partially vaccinated, 3,604 children received one dose of RV1, and 10,130 and 8,563 two and one dose of RV5, respectively (Table 2).

**Table 2:** **Patterns of vaccine administration in the rotavirus vaccinated cohort**

**Rotavirus vaccination**	**RV1**	**RV5**
**First dose**	**n = 24,723**	**n = 53,558**
Week of age at vaccination (median; IQR)	9 (9–11)	9 (9–10)
Dose administration >12 weeks^a^ (n; %)	5,263 (21%)	6,515(12%)
**Second dose**	**n = 21,119**	**n = 44,995**
Week of age at vaccination (median; IQR)	18 (17–19)	17 (15–18)
Dose administration >24 weeks^b^ (n; %)	1,172 (6%)	-
1^st^ - 2^nd^ dose interval in weeks (median; IQR)	9 (8–9)	8 (5–9)
1^st^ - 2^nd^ dose interval 22–27 days (n; %)	213 (1%)	1,162 (3%)
**Third dose**	**-**	**n = 34,865**
Week of age at vaccination (median; IQR)	-	26 (19–27)
Dose administration >32 weeks^c^ (n; %)	-	179 (1%)
2^nd^ – 3^rd^ dose interval in weeks (median; IQR)	-	8 (5–9)
2^nd^ – 3^rd^ dose interval 22–27 days (n; %)	-	1,338 (4%)

^a^>84 days; ^b^>168 days; ^c^>224 days.

### Rotavirus vaccine effectiveness

There were 22 probable or confirmed rotavirus hospitalizations among children fully vaccinated with RV1, 20 among children fully vaccinated with RV5, and 768 among non-rotavirus vaccinated children. Among cases vaccinated with RV1 and RV5, 22 (100%) and 19 (95%), respectively, had been vaccinated in accordance with the respective product’s indication. Adjusted VE against probable or confirmed rotavirus hospitalization was 86% (95% CI: 78-91%) for a full two-dose series of RV1, and 88% (95% CI: 81-92%) for a full three-dose series of RV5. Adjusted VE in children in full compliance with the product indication for timing of vaccine administration was 85% (95% CI: 77-90%) for RV1 and 87% (95% CI: 80-92%) for RV5.

There were six probable or confirmed rotavirus hospitalizations in children partially vaccinated with RV1, six in children vaccinated with two doses of RV5, and nine in children vaccinated with one dose of RV5. Unadjusted and adjusted assessments for all outcomes and vaccination status studied are shown in Table 3.

**Table 3:** **Unadjusted and adjusted rotavirus vaccine effectiveness in preventing hospitalizations due to rotavirus gastroenteritis by vaccine brand and number of doses received**

	**Confirmed and probable hospitalized rotavirus cases**			**Confirmed hospitalized rotavirus cases**		
**Vaccination status**	**Cases**	**VE** **(95%** **CI), %**		**Cases**	**VE** **(95%** **CI), %**	
		**Unadjusted**	**Adjusted**		**Unadjusted**	**Adjusted**
**Fully vaccinated**						
**RV1**	22	87(81–92)	86(78–91)	20	86(78–91)	84(75–90)
**RV5**	20	89(82–93)	88(81–92)	12	91(85–95)	91(84–95)
**RV1** ^**a**^	22	87(80–91)	85(77–90)	20	85(77–91)	84(74–90)
**RV5** ^**a**^	19	88(80–92)	87(80–92)	11	91(84–95)	91(83–95)
**Partially vaccinated**						
**RV1** (**1 dose**)	6	84(65–93)	82(60–92)	3	90(69–97)	89(66–97)
**RV5** (**2 doses**)	6	90(77–95)	89(75–95)	5	89(74–96)	89(73–95)
**RV5** (**1 dose**)	9	83(68–91)	83(66–91)	7	84(65–92)	84(66–92)
**Unvaccinated**						
	768	-	-	616	-	-

^a^Children fulfilling the product information criteria for timing of vaccination.

**Figure 2:**
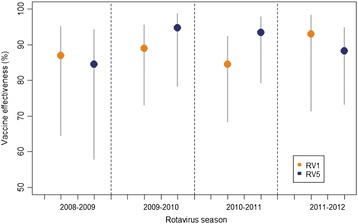
**Adjusted rotavirus vaccine effectiveness in preventing hospitalizations due to rotavirus gastroenteritis** (**confirmed or probable cases**) **among children fully vaccinated**, **by rotavirus season** (**1st December through 30th April**), **and by vaccine brand.**

There were no differences in adjusted VE between the four-rotavirus epidemic seasons studied (there were no cases in the vaccinated group during the season 2007–2008) among fully vaccinated children (Figure 2).

## Discussion

In this large population-based observational study including 78,281 children vaccinated with rotavirus vaccines and 96,643 children vaccinated with PCVs (and not with rotavirus vaccines), RV1 and RV5 showed high and similar effectiveness in preventing hospitalizations among young children. Our VE estimates are comparable to those obtained in other observational studies performed in countries in which rotavirus vaccines are included in the official schedules [13,26-28]. Our results are also similar to those obtained in post-licensure studies in countries without systematic rotavirus vaccination, including Spain [11,29,30]. Nevertheless, our study design, large sample size, and long study period, which included five rotavirus epidemic seasons, gave us the opportunity for providing precise adjusted effectiveness estimates by vaccine brand, including in partially vaccinated children, across a large time period.

Both vaccines were evaluated using a specific case definition. Since a discharge code may represent a rule-out diagnosis, an unconfirmed diagnosis, or may have been recorded incorrectly, we previously assessed the positive predictive value of the ICD-9-CM rotavirus code (008.61) using as gold standard the microbiological results from RedMIVA. The high positive predictive value found (≅90%) showed that hospitalized cases with or without a detection test result available could both be included, increasing the total number of study cases while assuring specificity in the case definition [31]. Accordingly, there were no significant differences between VE estimates based on confirmed rotavirus cases only and those including probable cases.

Natural immunity is acquired after early exposure to the virus and confers protection against subsequent infections. Although the protective effect of a natural infection is variable [3,4], the fact that rotavirus vaccines are not funded by the public health system, and must therefore be paid by parents, could have influenced the pediatrician’s recommendation to vaccinate children who suffered a prior rotavirus disease. By censoring children after the first confirmed rotavirus infection, we may have decreased the potential risk of confounding by indication [32]. Although we excluded children with prior known rotavirus infections, we could not be certain that all vaccinated children were naïve to rotavirus infections, because: (1) asymptomatic infections could provide protection similar to that induced by symptomatic disease [3], (2) our study began in 2007, year in which RedMIVA was still being implemented and not all laboratory results were available [22], and (3) an indeterminate number of ambulatory/Emergency Department cases were not tested for rotavirus infection. Nevertheless, these issues could not have substantially affected our results because the resulting bias should have driven results towards finding no differences between the groups being compared, and our VE estimates were high. Moreover, our results were comparable to those from other studies. Nonetheless, by censoring after ‘non-hospitalized’ infections, we might have introduced an informative censoring bias also affecting our VE estimates (these children are unlikely to subsequently have a rotavirus hospitalization). Given these concerns, we also performed an additional analysis without censoring children with non-hospitalized cases, and our VE estimates remained unchanged (results available in authors’ response to reviewers).

The fact that these vaccines were not included in the official immunization schedule might suggest the possibility of significant underreporting of vaccination, and differences between rotavirus vaccinees and non-vaccinees with respect to risk factors for natural disease. For these reasons, we chose a comparison cohort that included only children registered as having received another vaccine not included in the official immunization schedule, which should have minimized differences in the likelihood of vaccination registration and in socioeconomic conditions (a proxy for risk factors for disease and hospitalization). Despite using pneumococcal conjugate vaccinees as the comparison cohort, some residual socio-economical differences between groups could have remained because this vaccine is provided free of charge to a small group of children at high risk for complications of pneumococcal infection. The slight differences found between rotavirus and non-rotavirus vaccinees for having received at least one vaccine in a private center, and for having health coverage at the time of data extraction, could reflect in part the presence of this small group. Nevertheless, the differences found in VE between unadjusted and adjusted analyses were minimal, which suggests that the effect of confounding by socio-economical conditions, if any, was small. Also, since rotavirus and PCV are not included in the official immunization schedule, and are, therefore, administered mostly in private practices, our estimates could not be generalized to the overall Valencia population. Nonetheless, although the benefits of vaccination may be more important in higher risk groups, there is no biological reason to believe that the effectiveness of rotavirus vaccines within the same geographic region should differ by socio-economic conditions.

VE estimates were also high for partially vaccinated children. These findings were similar to other observational studies examining RV5 [28,33] and higher than those studying RV1 [34]. Because the vaccine is not funded by the public health system, there is a possibility of underreporting of rotavirus vaccination by providers. Nonetheless, according to a recent study, among all rotavirus vaccine doses distributed in Valencia during 2009–2012, most (86%) were registered in SIV as administered in children aged <1 year [35]. However, we cannot rule-out the possibility that some doses could have been missed or administered in private vaccination centers not using SIV, or outside the health care setting.

Another limitation might be due to the lack of systematic stool analyses among hospitalized children with acute gastroenteritis. Nonetheless, no differences in the proportions of laboratory tests performed were observed between the two cohorts. Moreover, as indicated earlier, the PPV of a hospital discharge diagnosis of rotavirus was high [31].

## Conclusions

In summary, RV1 and RV5 were over 85% effective in preventing rotavirus hospitalizations among young children living the Valencia Region, Spain, during the study period. The high effectiveness shown should contribute to decisions regarding the inclusion of rotavirus vaccines in the official immunization schedule in Spain. Additional information on rotavirus vaccine safety, duration of protection, and benefit-risk assessments in our setting will also be needed to inform such deliberations. This study also shows that Valencia’s databases could be used for the assessment of the effectiveness of vaccines not included in the official schedule.

## Abbreviations

CI: Confidence Interval
CMBD: Spanish hospital discharge database
HZ: Hazard ratio
IQR: Interquartile range
NHS: National Health System
PCV: Pneumococcal conjugate vaccine
RedMIVA: Valencia’s microbiological surveillance network database
RV1: Rotarix® (GlaxoSmithKline Biologicals, Rixensart, Belgium)
RV5: RotaTeq® (Merck & Co., Inc., West Point, PA, USA)
RVE: Rotavirus vaccine effectiveness
SIP: Valencia’s administrative population-based database
SIV: Valencia’s Vaccine Information System
VE: Vaccine Effectiveness
